# Dural Arteriovenous Fistula Associated With a Vestibular Tumor: An Unusual Case and Review of the Literature

**DOI:** 10.7759/cureus.2890

**Published:** 2018-06-27

**Authors:** Michael E Kritikos, Martin Oselkin, Nikhil Sharma, Pallavi P Gopal, Douglas C Bigelow, Sean Grady, Robert W Hurst, Bryan A Pukenas, Omar Choudhri, David Kung

**Affiliations:** 1 Neurosurgery, Hospital of the University of Pennsylvania, Philadelphia, USA; 2 Neuroradiology, Hospital of the University of Pennsylvania, Philadelphia, USA; 3 Pathology, Hospital of the University of Pennsylvania, Philadelphia, USA; 4 Otorhinolaryngology, Hospital of the University of Pennsylvania, Philadelphia, USA; 5 Radiology, Hospital of the University of Pennsylvania, Philadelphia, USA; 6 Neurosurgery, Hospital of University of the Pennsylvania, Philadelphia, USA

**Keywords:** dural arteriovenous fistula, vestibular schwannoma, octreotide scintigraphy, somatostatin receptor, angiogenesis, intracranial tumor

## Abstract

Intracranial dural arteriovenous fistulae (DAVF) are rare vascular malformations. They are generally considered to be acquired lesions, often attributed to dural sinus thrombosis and intracranial venous hypertension. The authors encountered a case of DAVF associated with an octreotide-positive vestibular schwannoma. A 46-year-old female had symptoms of right ear congestion accompanied by pulsatile tinnitus and mild hearing loss. Magnetic resonance imaging (MRI) identified a lobulated mass centered at the cerebellopontine angle. Preoperatively, on cerebral angiography, there was an incidental discovery of a DAVF in the right posterior fossa. The decision was made to proceed with resection of the tumor in a staged fashion. Her latest follow-up MRI showed no evidence of recurrent tumor. This is the second reported case of DAVF associated with an intracranial schwannoma. Findings are discussed along with a thorough review of the literature. This case, combined with the data from the literature review, led us to believe that tumor-related angiogenesis might contribute to DAVF formation.

## Introduction

Intracranial dural arteriovenous fistulae (DAVF) are abnormal direct shunts between dural arteries and dural venous sinuses, meningeal veins or cortical veins. They are located within or near the wall of the dural sinus and account for 10%-15% of all intracranial arteriovenous malformations [1]. DAVF primarily occur in adult patients and are most commonly located in the transverse-sigmoid sinus region followed by the cavernous sinus; however, any intracranial venous sinus may be involved [2]. They are widely considered to be acquired lesions. Their acquired nature is further supported by their association with dural sinus thrombosis, head trauma, intracranial or spinal tumors [3-4], localized infection, previous neurosurgical procedures [1-2], and hypercoagulation states [1].

We encountered a rare case of a transverse-sigmoid sinus DAVF associated with the presence of a vestibular schwannoma, which occluded the right transverse and sigmoid sinuses. The pre-operative diagnosis of this lesion was confounded by the fact that the tumor was positive on octreotide nuclear scintigraphy.

## Case presentation

**Figure 1:**
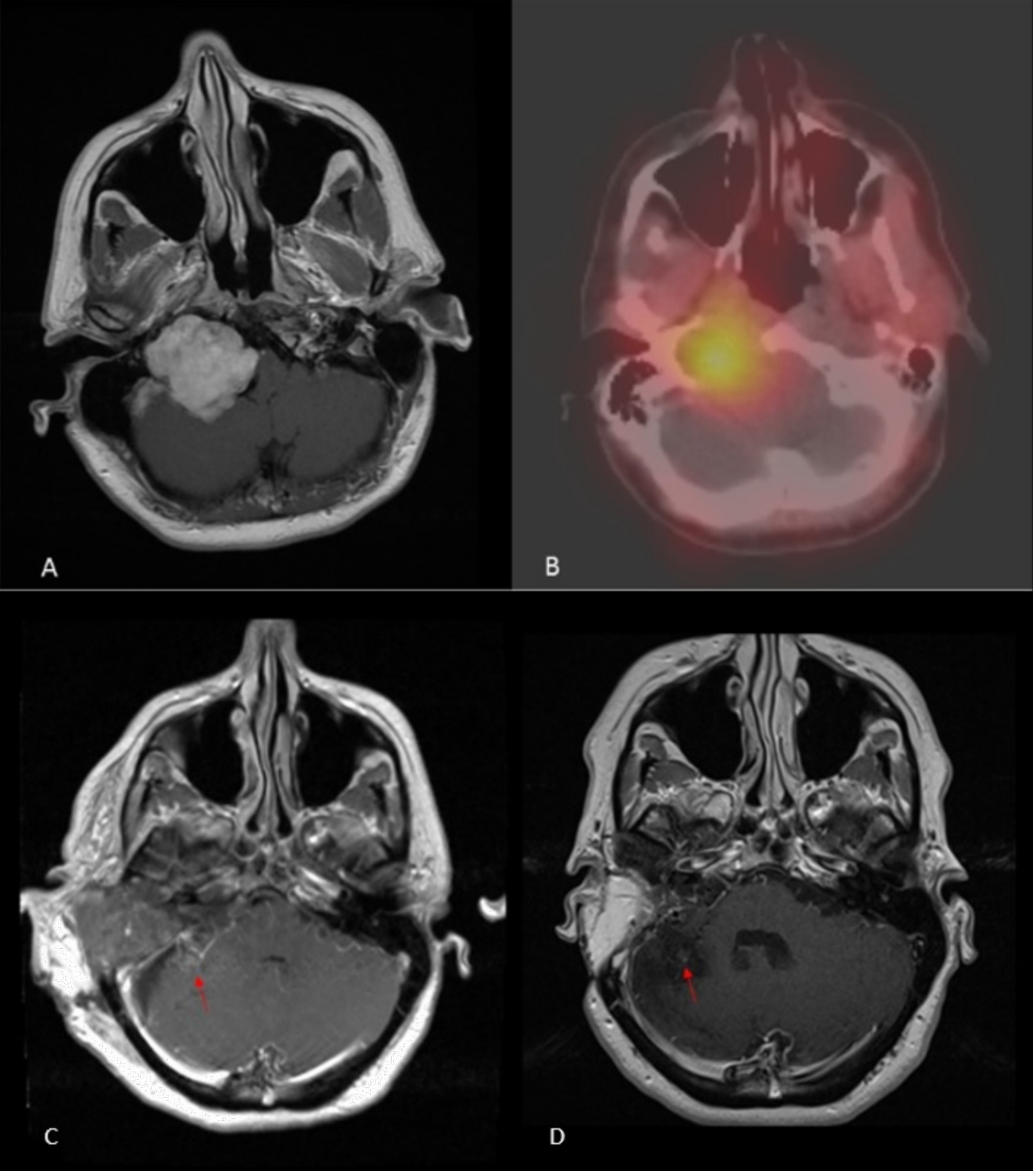
Magnetic resonance imaging and Octreoscan T1-weighted post-contrast MRI (magnetic resonance imaging) (A) demonstrates the avidly enhancing large right skull base mass centered at the jugular foramen, involving the right carotid space inferiorly and the right hypoglossal canal. 24-hour In-111 Pentetreotide SPECT-CT (single-photon emission computed tomography) fused images (B) demonstrates intense uptake in the mass. Initial post-operative T1-weighted post-contrast MRI (C) demonstrates expected post-surgical changes related to the skull base mass resection with no residual enhancing tumor. Follow-up MRI (D) four years later demonstrates stable post-operative changes with no residual mass. Tiny focus of enhancement beneath the tentorium likely reflected residual DAVF (dural arteriovenous fistula) (red arrow).

A 46-year-old female had symptoms of right ear congestion accompanied by pulsatile tinnitus and mild hearing loss. Neurological examination revealed weakness of cranial nerves X and XII. Magnetic resonance imaging (MRI) identified a 4.2 x 4.7 x 4.1 cm lobulated mass centered at the cerebellopontine angle that was hypointense on T1-weighted, heterogeneous on T2-weighted, and avidly enhancing on post-contrast images (Figure 1A). No significant component was noted within the internal auditory canal. An Octreoscan was performed, which showed intense increased tracer uptake in the mass centered in the region of the right jugular foramen, thereby supporting the suspected diagnosis of paraganglioma (Figure 1B).

**Figure 2:**
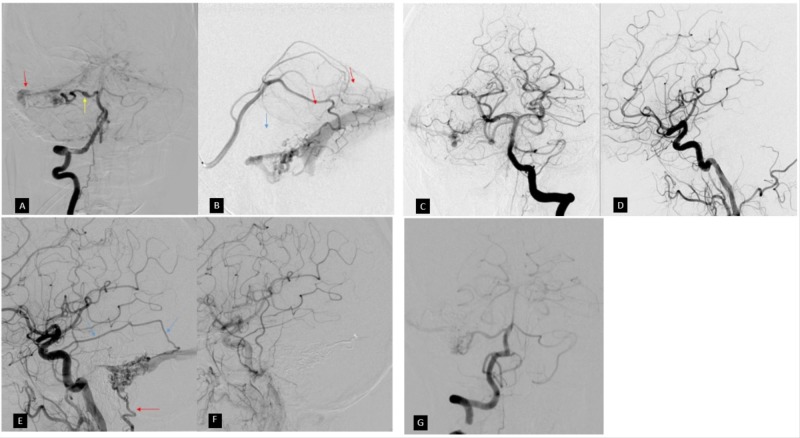
Digital subtraction angiography Initial cerebral angiogram planned for pre-operative embolization of the suspected paraganglioma did not demonstrate significant tumor vascularity. Incidentally, a dural arteriovenous fistula was seen predominantly supplied by the right AICA (anterior inferior cerebellar artery) on right vertebral artery injection (A). The right AICA is irregular with mild fusiform dilatation (yellow arrow). Microcatheter injection in the right SCA (superior cerebellar artery) (B) demonstrates a smaller component of the fistula not well seen on the right vertebral artery injection due to rapid shunting into the AICA. Additional very small arterial feeders were identified from branches of the right MMA (middle meningeal artery). There was cortical venous drainage (blue arrow) along the right cerebellar hemisphere to the right transverse sinus (red arrow), which then drained in retrograde fashion across the torcula and into the left transverse and sigmoid sinuses. Following embolization using Onyx in the right AICA and SCA DAVF (dural arteriovenous fistula) feeders, left vertebral artery DSA (digital subtraction angiogram) injection (C) demonstrates significant reduction in arteriovenous shunting. Again, there was no appreciable vascularity in the skull base mass. Right CCA (common carotid artery) injection (D) following embolization did not reveal additional feeders to the DAVF or mass. Follow-up DSA four years after initial embolization. Right CCA injection (E) reveals recurrence of the DAVF with new prominent feeders from the right MMA (blue arrow) and occipital (red arrow) arteries. Following embolization using Onyx in the right MMA feeder, right CCA injection in the late arterial phase (F) demonstrates resolution in shunting through the right CCA. (G) Right vertebral artery injection on the most recent DSA following MMA embolization demonstrates mild residual shunting similar to prior. There has been interval resolution of the dysplastic proximal right AICA.

Digital subtraction angiography (DSA) demonstrated a surprising lack of vascularity associated with the tumor. However, there was an incidental discovery of a Cognard IIa+b dural arteriovenous fistula in the right posterior fossa associated with an occluded right sigmoid sinus (Figure 2A-2D).

**Figure 3:**
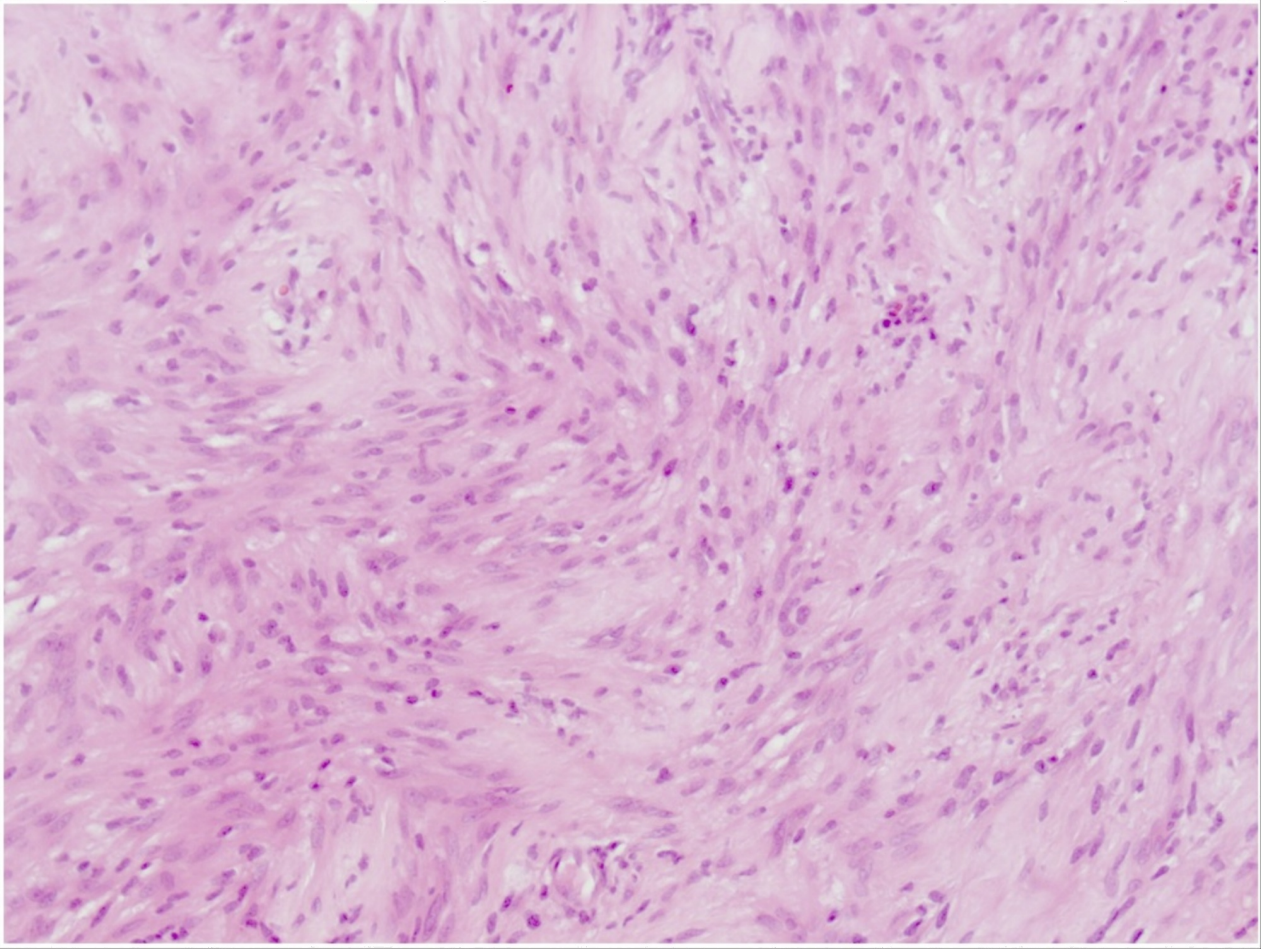
Photomicrograph Hematoxylin and eosin stained sections showed a biphasic tumor composed of spindle-shaped cells arranged in compact interlacing fascicles with areas of denser cellularity alternating with more loosely arranged cells, consistent with a schwannoma. Tumor cells were immune-positive for S100 (not shown). Areas of collagen deposition, ancient-type changes and lymphocytic perivascular infiltrates were also present (200x magnification).

Due to the extensive volume of tumor, the patient underwent a staged surgical resection. The first surgery included a modified radical right neck dissection, right superficial parotidectomy with facial nerve dissection, as well as the initial approach to the tumor and partial extradural resection. One week later, the patient underwent the second stage surgery where the tumor was radically resected using a right transcochlear approach. The right sigmoid sinus was ligated. The facial nerve was mobilized and transposed. Postoperative MRI confirmed complete resection of the tumor (Figure 1C). Histological analysis of samples from both surgeries confirmed the tumor not to be a paraganglioma, but instead a schwannoma (Figure 3).

She had a long recovery from the surgery due to the lower cranial nerve palsies but has shown gradual improvement. Her latest follow-up MRI (48 months from surgery) (Figure 1D) showed no evidence of recurrent tumor; however, there was some residual posterior fossa DAVF. At that time, she also reported bothersome pulsatile tinnitus that she perceives on the right despite a complete hearing loss on the right side. DSA revealed new large feeders from the right middle meningeal artery (MMA) and occipital arteries into the residual DAVF (figure 2E), which were not present on initial presentation (Figure 2D). The DAVF was embolized utilizing Onyx in the right MMA. Final right common carotid artery (CCA) angiogram demonstrated resolution in arteriovenous shunting (Figure 2F). Vertebral artery injection after embolization demonstrated minimal residual shunting via the right anterior inferior cerebellar artery (AICA) (Figure 2G). A further intervention was declined by the patient and angiographic follow-up in one year has been scheduled.

## Discussion

In our patient, we encountered a transverse-sigmoid sinus DAVF associated with a schwannoma of the right skull base. Although angiography was not available prior to diagnosis of the large skull base mass, one could hypothesize that the schwannoma led to the venous sinus obstruction, and subsequently the formation of the DAVF. DAVF formation attributed to a tumor has been described, but it is rare. A detailed PubMed query was performed, which identified 26 previously reported cases (Table 1) [3-17]. Analysis of the previously reported cases exhibited a slight female predominance (61.5%) and a mean age of 60.26 years (range 25-78). This is only the second case associated with an intracranial schwannoma. In the vast majority of cases (78%), the tumor was a meningioma. Although there are exceptions, most cases of DAVF associated with tumor are also associated with sinus obstruction, underlining the importance of venous outflow obstruction in DAVF pathophysiology. Specifically, the most common location of occlusion was the sigmoid sinus, which was occluded in 38.5% of cases. Interestingly, 23% of cases were not related to sinus occlusion. The most common location for a DAVF associated with a tumor was the transverse-sigmoid sinus (48% of cases). Some investigators hypothesize that meningiomas are the most likely tumor type to be associated with DAVF due to their highly vascular nature, which in turn accentuates venous hypertension, especially when the sinus is occluded [14].

**Table 1:** Reported cases of dural arteriovenous fistulae associated with tumor in adult patients

Author (year)	Age/Sex	Tumor/Location	DAVF	Sinus Occlusion	Treatment of DAVF	Follow-up
Sawamura et al. (1991) [7]	68/M	Meningioma/R occipital (parafalcine)	Tentorium	Unclear	Unclear (tumor surgical resection)	None reported
Yokota et al. (1993) [8]	65/F	Meningioma/R sigmoid sinus groove	R transverse sinus	R sigmoid sinus	Pre-op TAE and surgical packing	36 mo (disappeared)
Yamakami et al. (1998) [9]	69/M	Schwannoma/R jugular foramen	R sigmoid sinus	R sigmoid sinus	Pre-op TAE and surgical resection	None (disappeared post-op)
Arnautovic et al. (1998) [3]	63/F	Meningioma/L tentorium	L transverse and sigmoid sinuses	L transverse sinus	Pre-op TAE and surgical resection	40 mo (disappeared)
	57/F	Paraganglioma/R jugular foramen	R transverse and sigmoid sinuses	R sigmoid sinus	Pre-op TAE and surgical resection	28 mo (disappeared)
Chung et al. (1999) [10]	62/F	Meningioma/R tentorium	R transverse sinus	R transverse and sigmoid sinuses	None (tumor subtotal surgical resection)	None (disappeared post-op)
Vilela et al. (2001) [6]	51/M	Meningioma/ Parasagittal	2 DAVFs: R superior petrosal sinus AND R parietal convexity	Superior sagittal sinus	Surgical disconnection of DAVFs w/o tumor resection	60 mo (no DAVF recurrence)
	56/ Unspecified	Meningioma/R skull base	R transverse sinus	R sigmoid sinus	None (Pre-op tumor TAE and tumor surgical resection)	24 mo (clinically stable)
Horinaka et al. (2003) [11]	69/F	Meningioma/R transverse sinus	R transverse sinus	R transverse sinus and part of sigmoid sinus	Transverse sinus resection (part of tumor resection surgery)	9 mo (disappeared)
Ahn et al. (2003) [5]	45/F	Meningioma/L parietal convexity	L transverse and sigmoid sinuses	L sigmoid sinus (partial, w/o venous congestion)	None (Pre-op tumor TAE and tumor surgical resection)	12 mo (disappeared)
Inoue et al. (2007) [12]	76/M	Meningioma/L sphenoidal ridge	R cavernous sinus	None	TVE (followed by tumor surgical resection)	12 mo (disappeared)
Zhou et al. (2007) [13]	42/M	Meningioma/L anterior clinoid (tumor unnoticed pre-op)	L spheno-basilar sinus	None	Pre-op TAE and surgical disconnection	24 mo (disappeared)
Toledo et al. (2010) [14]	60/M	Meningioma/L parietal lobe (parafalcine)	Superior sagittal sinus	Superior sagittal sinus	Pre-op TAE and surgical occlusion	None (disappeared post-op)
Kalani et al. (2011) [15]	25/F	Hemangiopericytoma/L parieto-occipital (parafalcine)	Multiple DAVFs: Unclear locations (one torcular DAVF)	Superior sagittal sinus	Pre-op TAE and surgical disconnection (during tumor surgical resection)	7 mo (patient asymptomatic but has residual DAVF)
Enatsu et al. (2012) [16]	76/F	Meningioma/R tentorium	R transverse sinus	R transverse and sigmoid sinuses	Pre-op TAE and intra-op feeder coagulation and occluded sinus resection (during tumor surgical resection)	1 mo (disappeared)
Vellimana et al. (2014) [17]	55/M	Meningioma/L sphenoid wing/orbit	2 DAVFs: both L convexity	None	None (tumor subtotal surgical resection)	None reported
	73/F	Meningioma (radiologic diagnosis) /L occipital/tentorium	L cavernous sinus	L cavernous sinus and L transverse sinus (partial)	TAE and TVE followed by GK (no tumor resection)	None reported
	69/M	Meningioma/L convexity (fronto-temporal)	R transverse sinus	Partial of multiple unspecified sinuses	None (TAE of tumor followed by tumor surgical resection)	None reported
	41/F	Meningioma/R lateral ventricle-trigone	L convexity	None	TAE (followed by tumor surgical resection)	None reported
	54/F	Meningioma (radiologic diagnosis)/R petrous apex	L transverse and sigmoid sinuses	R cavernous sinus	TAE (followed by GK for tumor)	None reported
	57/M	Meningioma (radiologic diagnosis)/L parasellar	L convexity	L cavernous sinus	None (fractionated radiotherapy for tumor)	None reported
	78/F	Meningioma (radiologic diagnosis)/L convexity (frontal)	L cavernous sinus	L cavernous sinus	TAE (no tumor resection)	None reported
	67/F	Phosphaturic mesenchymal tumor/L anterior cranial fossa	Lateral tentorial	None	Surgical resection (following failed TAE)	None reported
	72/M	Meningioma/ Planum sphenoidale	Ethmoidal sinus	None	Surgery (during tumor resection)	None reported
	61/F	Meningioma/ Olfactory groove	R transverse and sigmoid sinuses	Superior sagittal sinus	GK and TAE	None reported
Hatanaka et al. (2015) [4]	70/F	Schwannoma/ Spinal (C2 level)	R transverse and sigmoid sinuses	R sigmoid sinus	TAE and TVE (followed by tumor surgical resection). GK after DAVF recurrence 6 mo post-intervention	33 mo (disappeared)
DAVF: dural arteriovenous fistula; TAE: transarterial embolization; TVE: transvenous embolization; GK: gamma knife radiosurgery						

Another unique characteristic in our case was the positive 111In-octreotide scintigraphy (Octreoscan), which is used to evaluate the presence of somatostatin receptors in intracranial tumors in vivo. Expression of somatostatin receptors has been linked with increased angiogenic activity in meningiomas and other neoplasias [18]. This is only the second reported case of an intracranial schwannoma with positive octreotide scintigraphy [19]. Past studies have shown that somatostatin receptors are expressed in most meningiomas, paragangliomas and hemangiopericytomas, but not in schwannomas, with some investigators using octreotide scintigraphy for the differential diagnosis of meningioma versus schwannoma [19-20]. Our case further supports the conclusion by Dupuch et al. that no investigation is able to establish a definitive preoperative differential diagnosis between schwannoma and meningioma [19]. Taking into consideration the case we encountered, we hypothesize that aberrant angiogenesis through somatostatin receptor pathways may play a role in the pathogenesis of DAVF associated with intracranial tumors. Even though there is currently no evidence showing a direct connection between somatostatin pathways and DAVF formation, studies have shown that the presence of somatostatin receptors correlates with aberrant angiogenesis in various tumor settings [18]. Our hypothesis aims at opening new avenues of investigation that will help in better understanding DAVF pathophysiology.

## Conclusions

In summary, we present an unusual case of DAVF associated with an octreotide positive vestibular schwannoma. This case demonstrates that lesions in the cerebellopontine angle could affect local vasculature, possibly by both causing local venous hypertension and somatostatin receptor-related angiogenesis, to the extent it may lead to a DAVF formation.
